# Root canal morphometric indices of anterior teeth and their association with chronological age: a retrospective CBCT study in an Iranian population

**DOI:** 10.1186/s12903-026-09866-3

**Published:** 2026-09-16

**Authors:** Nazanin Zargar, Arefeh Shamsi, Yaser Safi, Alireza Akbarzadeh Baghban, Asal Fetrati, Payam Paymanpour

**Affiliations:** 1 Department of Endodontics, School of Dentistry, Shahid Beheshti University of Medical Sciences, Tehran, Iran; 2 School of Dentistry, Shahid Beheshti University of Medical Sciences, Tehran, Iran; 3 Department of Oral and Maxillofacial Radiology, School of Dentistry, Shahid Beheshti University of Medical Sciences, Tehran, Iran; 4 Proteomics Research Center, Department of Biostatistics, School of Allied Medical Sciences, Shahid Beheshti University of Medical Sciences, Tehran, Iran; 5 Department of Oral and Maxillofacial Surgery, School of Dentistry, University of Michigan, Ann Arbor, MI USA; 6 Department of Prosthodontics, Louisiana State University Health Science Center School of Dentistry, New Orleans, LA USA

**Keywords:** Age estimation, Anterior teeth, Cone-beam computed tomography, Dental age estimation, Forensic dentistry, Morphometric indices, Root canal morphology

## Abstract

**Background:**

This study examined the associations between root canal morphometric indices of anterior teeth and chronological age and evaluated their apparent model fit for age association analysis in an Iranian population using cone-beam computed tomography (CBCT).

**Methods:**

In this retrospective cross-sectional study, 1,200 intact anterior teeth from 100 patients were analyzed using CBCT images. Although each patient contributed 12 anterior teeth, regression analyses were performed separately for each tooth type. Participants were evenly distributed across five predefined age strata (15–24, 25–34, 35–44, 45–54, and ≥ 55 years), while age was treated as a continuous variable in all analyses. Measurements included length ratio (LR), buccolingual and mesiodistal width ratios (WR), and canal shape ratio (CSR), obtained using OnDemand3D™ software. Multiple linear regression was used to assess age associations and construct exploratory age‑estimation models, while logistic regression was used for exploratory sex‑prediction analyses.

**Results:**

The sample comprised 69% females and 31% males, with a mean age of 39.65 ± 14.58 years. Several morphometric indices showed tooth‑ and level‑specific associations with age. Buccolingual WR at the cementoenamel junction (CEJ) demonstrated the most frequent association. CSR showed significant negative associations with age in maxillary and mandibular central incisors and mandibular lateral incisors. LR showed only limited association with age. Sex demonstrated limited and inconsistent relationships. The maxillary right central incisor showed the strongest apparent model fit (R² = 0.465), with buccolingual WR at the CEJ as the most influential morphometric index across models.

**Conclusion:**

Root canal morphometric indices exhibit tooth-specific associations with chronological age in this population. Central incisors and mandibular lateral incisors demonstrated the most consistent age-related associations, and buccolingual WR at the CEJ showed the most frequent association with age. Tooth‑specific variability in model fit suggests that morphometric behavior, rather than methodological factors, drives the strength of age‑related associations. These findings represent exploratory age‑estimation patterns.

**Supplementary Information:**

The online version contains supplementary material available at https://doi.org/10.1186/s12903-026-09866-3.

## Background

As part of the natural physiological aging process, secondary dentin formation by odontoblasts—highly specialized, terminally differentiated post-mitotic cells of ectomesenchymal origin—continues throughout life following tooth eruption and functional occlusion, progressively altering root canal morphology through continued dentin deposition [1]. This sustained deposition of secondary dentin along the pulpal walls results in a gradual reduction of pulp space with advancing age, reflecting cumulative age-related changes within the dentin–pulp complex [2]. These changes provide a biological basis for adult age estimation, as progressive reductions in pulp dimensions have been consistently shown to correlate with chronological age [3, 4]. In the context of dental trauma, deviations from expected age-related morphometric patterns—due to disruption of pulpal blood supply—may provide indirect insight into the timing of injury [5]. Because dental tissues are relatively resistant to mechanical, chemical, and thermal degradation and are less influenced by systemic disease, drug intake, or endocrine alterations than skeletal tissues, they constitute particularly robust substrates for chronological age estimation in forensic and medico-legal contexts [6].

Beyond their forensic relevance, these morphologic alterations have substantial clinical implications in endodontics, as they influence canal anatomy, treatment planning, instrumentation strategies, and procedural complexity [7]. Accordingly, root canal morphometric data contribute to a more nuanced understanding of internal anatomical dynamics and may assist in selecting appropriate cleaning and shaping techniques to optimize treatment outcomes [8, 9].

The advent of cone-beam computed tomography (CBCT) has significantly advanced dental imaging by enabling high-resolution, three-dimensional visualization of tooth structure, and internal anatomy in vivo [10, 11]. Compared with conventional two-dimensional radiography, CBCT provides multiplanar reconstructions that reduce superimposition and geometric distortion while allowing for more accurate assessment of root and canal morphology [12, 13]. Importantly, CBCT delivers these detailed anatomical insights with substantially lower radiation exposure than conventional medical computed tomography, making it a clinically acceptable and safer imaging option when justified [11, 13].

Although three-dimensional pulp–tooth volumetric analysis is conceptually appealing, its practical implementation in CBCT-based age estimation remains methodologically demanding. Volumetric approaches are highly dependent on segmentation accuracy, voxel size, threshold selection, and software performance, factors that can introduce variability and limit reproducibility, particularly in retrospective datasets [3, 14, 15]. In contrast, ratio-based linear and cross-sectional morphometric indices offer several pragmatic advantages. These metrics are faster to obtain, require fewer segmentation steps, and rely on easily identifiable anatomical landmarks, resulting in higher intra- and inter-examiner reproducibility across studies [3, 15]. Importantly, systematic reviews and meta-analyses of CBCT-based dental age estimation have demonstrated that volumetric methods do not consistently outperform metric or sectional approaches, with comparable correlation coefficients and predictive performance reported for both methodologies. Moreover, ratio-based measurements inherently reduce the effects of magnification, angular distortion, and unit variability, facilitating standardization and cross-study comparability across imaging systems [15]. Given the modest or inconsistent predictive gains of volumetric models relative to their substantially greater time requirements and methodological burden, sectional morphometric ratios represent a defensible, efficient, and robust alternative for large-scale retrospective analyses and routine forensic applications, while still capturing the biologically relevant effects of secondary dentin deposition [3].

Anterior teeth are optimal targets for CBCT-based morphometric age estimation because of their relatively simple root canal anatomy, high long-term retention, and favorable measurement reproducibility [16, 17]. However, growing evidence indicates that dental age estimation models exhibit population-specific variability, underscoring the need for population-tailored validation [18]. Despite the methodological advantages of ratio-based morphometric indices in CBCT analysis, data characterizing their age-related behavior in Iranian populations remain limited. Accordingly, this retrospective cross-sectional study investigated the associations between anterior tooth root canal morphometric indices and chronological age and evaluated their performance for age estimation in an Iranian population using CBCT.

## Methods

### Study design

This retrospective cross-sectional study was approved by the Ethics Committee of Shahid Beheshti University School of Dentistry (Ethics Code: IR.SBMU.DRC.REC.1402.061), and all patients provided written informed consent for the use of their imaging data for research purposes. For participants younger than 16 years of age, written informed consent was obtained from a parent or legal guardian. The study was conducted in accordance with the Declaration of Helsinki and relevant institutional guidelines. The study was designed, conducted, and reported in accordance with the Strengthening the Reporting of Observational Studies in Epidemiology (STROBE) guidelines.

### Image selection

**Fig. 1:**
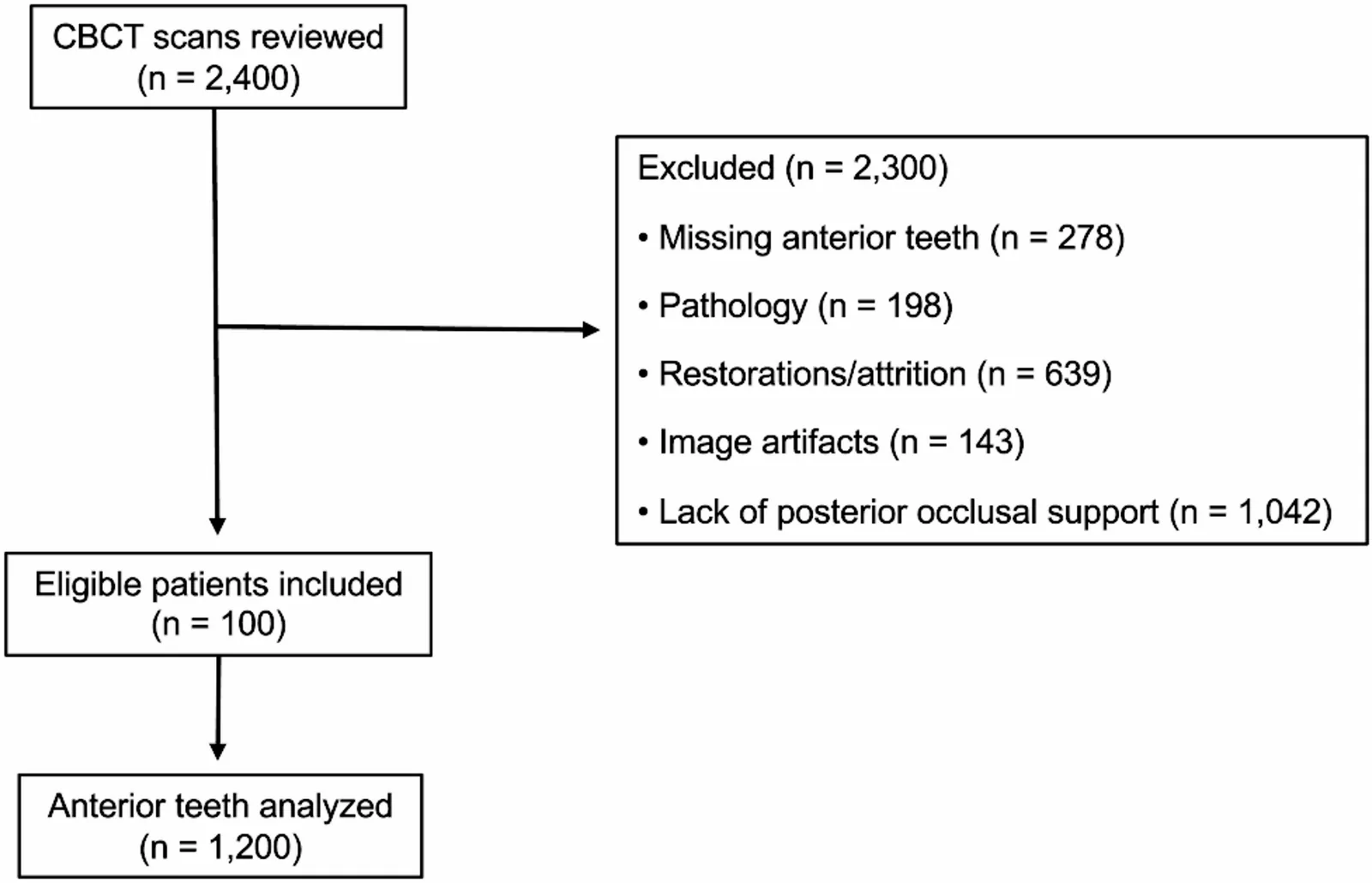
Flow diagram of the CBCT scan selection process. Of the 2,400 CBCT examinations screened, 2,300 were excluded according to predefined eligibility criteria. A total of 100 patients met the inclusion criteria and were analyzed, yielding 1,200 anterior teeth for morphometric evaluation

The CBCT archive of the Radiology Department at Shahid Beheshti University School of Dentistry was retrospectively reviewed, including all scans acquired between September 2020 and September 2023. Patients were selected according to the predefined inclusion and exclusion criteria described in the following subsection. Of the 2,400 available CBCT scans, 100 patients satisfied these criteria and were included in the study. Because all anterior teeth were required to fulfill the eligibility criteria, all 12 anterior teeth from each included patient were analyzed, yielding a total of 1,200 teeth for morphometric evaluation. The patient selection and tooth inclusion process are summarized in Fig. 1. All included scans had been obtained using a NewTom VGI unit (Verona, Italy) with standardized acquisition parameters: 110 kVp, automatic mA, standard resolution, a 6 × 6 cm field of view, voxel size of 150 μm, and exposure time of 6 seconds.

### Inclusion and exclusion criteria

The inclusion criteria required the presence of all fully developed, intact maxillary and mandibular anterior teeth, with no signs of attrition, caries, periapical pathology, root resorption, dental anomalies, or malposition. Scans with artifacts or insufficient image quality were excluded. Patients lacking posterior occlusal support were also excluded.

### Age groups and sample size selection

Participants were evenly distributed across five predefined age strata (15–24, 25–34, 35–44, 45–54, and ≥ 55 years) to ensure balanced sampling. Age was analyzed as a continuous variable (mean ± SD: 39.65 ± 14.58 years) in all statistical models. Sample size estimation was based on Kaya et al. [7] and calculated using G*Power software (version 3.1.9.7; Heinrich Heine University Düsseldorf, Germany), assuming a 95% confidence level and 80% statistical power. A minimum of 20 teeth per age stratum was estimated to provide adequate power for detecting moderate age-related associations in morphometric indices.

### Root canal morphometric measurements

**Fig. 2:**
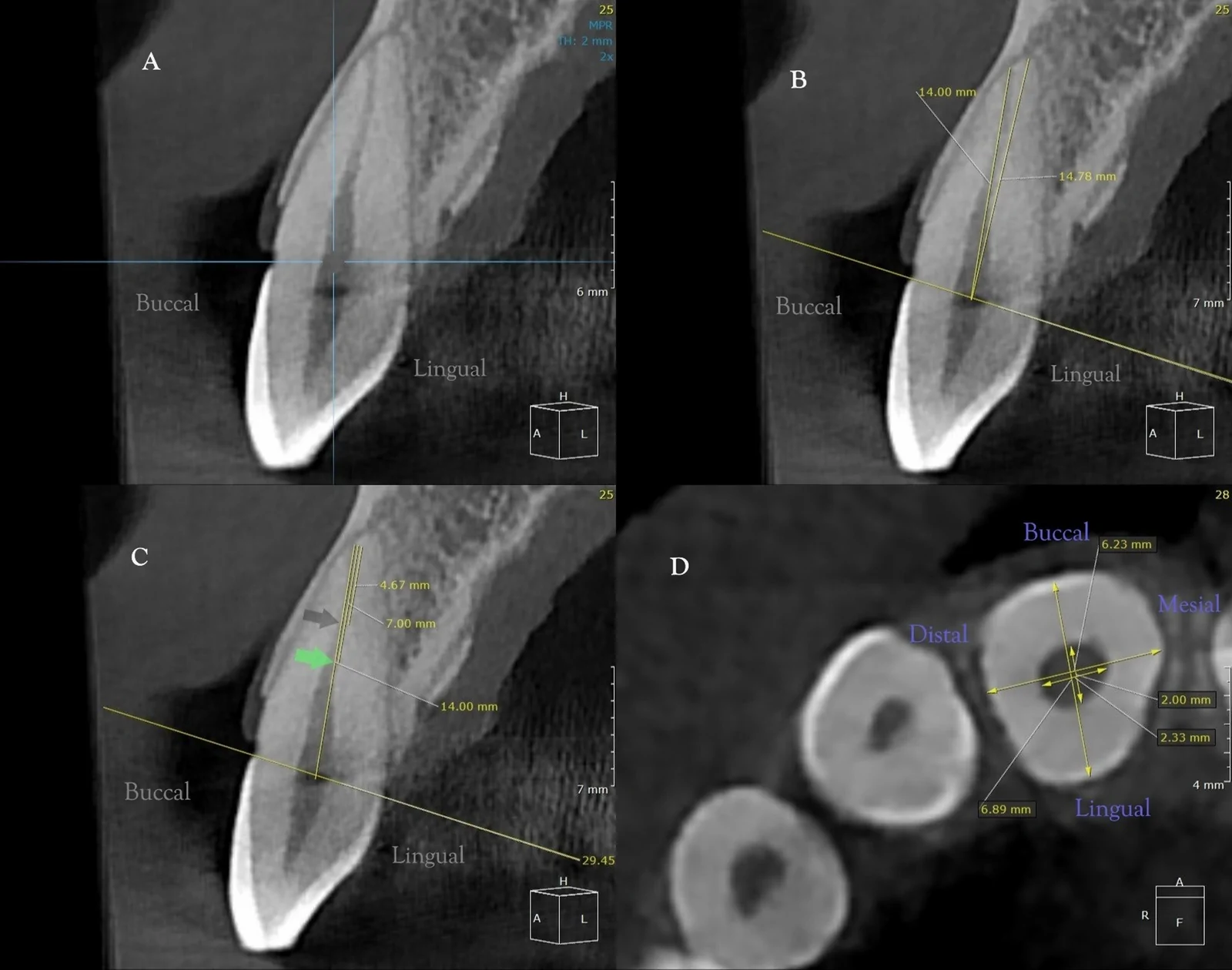
Stepwise CBCT measurement protocol for root canal morphometrics. **A** Standardized midsagittal view of the maxillary right central incisor (slice thickness = 2.0 mm) oriented parallel to the long axis of the tooth. **B** Root and canal length measurement from the CEJ line (connecting the most cervical extents of the buccal and lingual enamel); root length (14.78 mm) is measured as the distance from the radiographic apex to the CEJ line, while canal length (14.00 mm) is measured from the midpoint of the root canal boundaries at the CEJ level along the longitudinal axis to the apical foramen. **C** Establishment of cross-sectional measurement levels based on canal length as: the CEJ level, the mid-root level (green arrow; 50% of root length from CEJ, 7.00 mm), and the apical level (gray arrow; two-thirds of root length from CEJ, 4.67 mm from apex). **D** Representative axial cross-section (slice thickness = 0.2 mm) generated perpendicular to the root long axis (shown at the CEJ level) illustrating buccolingual and mesiodistal root and canal width measurements used to calculate width ratios (WR) and canal shape ratios (CSR)

DICOM files were imported into OnDemand3D™ software (Cybermed Inc., Seoul, South Korea; version 10.0.1) using lossless compression. Multiplanar reconstructions (MPR) were aligned parallel to the long axis of each tooth from which the midsagittal view (slice thickness = 2 mm) was used to establish longitudinal landmarks (Fig. 2). The cementoenamel junction (CEJ) level was defined by a line connecting the most cervical extents of the enamel on the buccal and lingual surfaces. Root length was determined as the distance from the radiographic apex to the CEJ line. Canal length was measured from the apical foramen to the midpoint between the buccal and lingual boundaries of root canal at the CEJ level. The 2 mm slice thickness was chosen to optimize the signal-to-noise ratio and ensure consistent visual delineation of the canal boundaries against surrounding dentin across multiplanar reorientations, thereby minimizing local artifact distortions.

Subsequently, axial cross-sections (slice thickness = 0.2 mm) were generated perpendicular to the root long axis at the CEJ level, mid-root level (50% of the canal length), and the apical level (apical one-third of the canal length).

Length ratio (LR; canal length divided by root length) and width ratio (WR; canal width divided by root width) were calculated as follows:

$$LR=canal\:length\:/\:root\:length$$

$$WR=canal\:width\:/\:root\:width$$

WR was measured in both buccolingual (BL) and mesiodistal (MD) dimensions at the CEJ, mid-root, and apical third (Fig. 2).

A canal shape ratio (CSR), representing the proportional canal shape at each level, was defined as:

$$CSR=WR_{\left(BL\right)}\:/\:WR_{\left(MD\right)}$$

All measurements were performed by a single examiner blinded to chronological age. Intra-examiner reliability was assessed by remeasuring 15 randomly selected scans after four weeks, and intraclass correlation coefficients were calculated to quantify consistency.

### Statistical analysis

Descriptive statistics were used for morphometric indices and demographic variables. Continuous variables were summarized as means and standard deviations.

Multiple linear regression analyses were performed separately for each tooth type to assess associations between root canal morphometric indices and age (treated as a continuous variable). Associations between morphometric indices and sex were also explored using multiple linear regression. In these models, morphometric indices served as dependent variables, with sex and age entered as independent variables.

As for the age estimation analysis, stepwise procedure was used; age and morphometric indices were specified as the dependent and independent variables, respectively. Given the exploratory objective of the study, these models were intended to identify tooth‑specific associations rather than to develop validated or generalizable age‑estimation models.

In a separate exploratory analysis, sex was modeled as a dependent variable in multiple logistic regression using a forward stepwise (likelihood ratio) procedure. Because these analyses were exploratory and no correction for multiple testing was applied, the resulting sex‑prediction models were interpreted cautiously and not considered as definitive predictive models.

Collinearity among candidate predictors in multiple regression models was evaluated using variance inflation factor (VIF) and tolerance values. Because each regression analysis was performed separately for each tooth type, each model included one observation per participant. Therefore, the tooth-specific regression models were based on independent participant-level observations rather than pooled tooth-level data. All statistical analyses were performed using SPSS software (version 26.0; IBM Corp., Armonk, NY, USA). Statistical significance was set at *P* < 0.05 for all inferential analyses.

## Results

Participant characteristics are summarized in Table 1. Descriptive statistics for morphometric indices across anterior teeth are presented in Table 2. Intra-examiner reliability was excellent, with intraclass correlation coefficients (ICCs) exceeding 0.97 for all measured variables.

**Table 1:** Distribution of participants by age group and sex (*n* = 100)

Age Group (years)	Female	Male	Total
15–24	14	6	20
25–34	15	5	20
35–44	12	8	20
45–54	15	5	20
≥ 55	13	7	20
Total	69	31	100

**Table 2:** Mean (SD) of root canal morphometric indices for anterior teeth (*n* = 100)

**Morphometric Index**	**Maxillary**						**Mandibular**					
	**Right**			**Left**			**Right**			**Left**		
	**C**	**LI**	**CI**	**CI**	**LI**	**C**	**C**	**LI**	**CI**	**CI**	**LI**	**C**
LR	0.997 (0.023)	0.983 (0.021)	0.985 (0.025)	0.983 (0.028)	0.982 (0.025)	0.999 (0.026)	0.986 (0.022)	0.979 (0.023)	0.974 (0.024)	0.974 (0.025)	0.979 (0.022)	0.990 (0.024)
WR at CEJ (BL)	0.275 (0.056)	0.211 (0.064)	0.194 (0.058)	0.197 (0.060)	0.219 (0.066)	0.284 (0.064)	0.277 (0.055)	0.215 (0.066)	0.210 (0.069)	0.211 (0.074)	0.221 (0.063)	0.291 (0.060)
WR at CEJ (MD)	0.185 (0.053)	0.233 (0.083)	0.282 (0.073)	0.275 (0.080)	0.248 (0.068)	0.192 (0.057)	0.157 (0.057)	0.204 (0.095)	0.200 (0.099)	0.221 (0.103)	0.221 (0.099)	0.165 (0.060)
CSR at CEJ	1.612 (0.645)	1.033 (0.497)	0.761 (0.364)	0.794 (0.363)	0.974 (0.471)	1.625 (0.669)	1.995 (0.821)	1.315 (0.759)	1.416 (0.991)	1.225 (0.746)	1.257 (0.710)	1.983 (0.787)
WR at mid-root (BL)	0.167 (0.055)	0.175 (0.046)	0.162 (0.040)	0.160 (0.043)	0.186 (0.045)	0.185 (0.059)	0.189 (0.069)	0.241 (0.092)	0.223 (0.085)	0.226 (0.082)	0.237 (0.091)	0.204 (0.079)
WR at mid-root (MD)	0.214 (0.079)	0.208 (0.069)	0.222 (0.075)	0.230 (0.075)	0.214 (0.066)	0.196 (0.058)	0.180 (0.058)	0.187 (0.068)	0.201 (0.085)	0.188 (0.076)	0.209 (0.088)	0.188 (0.068)
CSR at mid-root	0.896 (0.531)	0.937 (0.439)	0.807 (0.307)	0.784 (0.323)	0.951 (0.357)	1.037 (0.549)	1.200 (0.700)	1.491 (0.843)	1.336 (0.839)	1.377 (0.681)	1.363 (0.848)	1.230 (0.683)
WR at apical third (BL)	0.169 (0.051)	0.185 (0.060)	0.166 (0.048)	0.166 (0.044)	0.190 (0.054)	0.185 (0.051)	0.181 (0.056)	0.198 (0.076)	0.182 (0.060)	0.199 (0.073)	0.201 (0.075)	0.177 (0.066)
WR at apical third (MD)	0.225 (0.072)	0.236 (0.066)	0.243 (0.088)	0.252 (0.089)	0.232 (0.070)	0.214 (0.062)	0.196 (0.062)	0.218 (0.068)	0.218 (0.072)	0.216 (0.072)	0.215 (0.069)	0.209 (0.071)
CSR at apical third	0.827 (0.367)	0.855 (0.381)	0.778 (0.369)	0.744 (0.318)	0.903 (0.380)	0.951 (0.442)	1.011 (0.444)	1.053 (0.687)	0.926 (0.428)	1.036 (0.555)	1.035 (0.540)	0.970 (0.597)

*BL* Buccolingual, *MD* Mesiodistal, *CEJ* Cementoenamel junction, *C* Canine, *LI* Lateral incisor, *CI* Central incisor, *LR* Length ratio, *WR* Width ratio, *CSR* Canal shape ratio

### Age associations (Table 3)

Buccolingual WR at the CEJ was the index most frequently associated with age, demonstrating predominantly negative associations across all teeth (*p* ≤ 0.05). Mesiodistal WR was generally positively associated with age (*p* ≤ 0.05), except at the CEJ of the right canines, where a significant negative relationship was observed (*p* < 0.05).

**Table 3:** Associations between age and morphometric indices in anterior teeth. Values represent unstandardized regression coefficients (B) from linear regression models, indicating change per year of age. Direction of association is indicated by (+) or (−)

**Morphometric Index**	**Maxillary**						**Mandibular**					
	**Right**			**Left**			**Right**			**Left**		
	**C**	**LI**	**CI**	**CI**	**LI**	**C**	**C**	**LI**	**CI**	**CI**	**LI**	**C**
LR	ns	ns	0.0004 *(-)	ns	0.0004 *(-)	ns	ns	ns	ns	ns	ns	ns
WR at CEJ (BL)	0.001 *(-)	0.002 **(-)	0.002 **(-)	0.002 **(-)	0.002 *(-)	ns	0.001 * (-)	0.003 * (-)	0.003 ***(-)	0.003 ***(-)	0.003 *(-)	0.001 *(-)
WR at CEJ (MD)	0.001 *(-)	ns	0.001 *(+)	ns	ns	ns	0.001 *(-)	0.002 *(+)	0.002 *(+)	0.003 ***(+)	0.003 ***(+)	ns
CSR at CEJ	ns	ns	0.010 ***(-)	0.009 ***(-)	ns	ns	ns	0.020 ***(-)	0.022 *(-)	0.024 ***(-)	0.020 ***(-)	ns
WR at mid-root (BL)	0.001 *(-)	ns	0.001 ***(-)	0.001 ***(-)	ns	ns	0.001 *(-)	0.002 *(-)	0.001 *(-)	ns	0.002 *(-)	ns
WR at mid-root (MD)	ns	ns	0.002 *(+)	0.001 *(+)	ns	ns	ns	0.001 *(+)	0.002 *(+)	0.002 ***(+)	0.002 *(+)	ns
CSR at mid-root	ns	ns	0.010 ***(-)	0.008 ***(-)	ns	ns	ns	0.013 *(-)	0.016 *(-)	0.014 *(-)	0.022 ***(-)	ns
WR at apical third (BL)	ns	ns	0.002 ***(-)	0.002 ***(-)	ns	ns	ns	0.002 ***(-)	0.001 *(-)	0.001 *(-)	0.001 *(-)	0.001 *(-)
WR at apical third (MD)	0.002 *(+)	0.001 *(+)	0.002 *(+)	0.002 *(+)	ns	ns	ns	0.002 ***(+)	0.001 *(+)	0.001 *(+)	0.002 ***(+)	ns
CSR at apical third	ns	ns	0.010 ***(-)	0.010 ***(-)	ns	ns	ns	0.016 *(-)	0.009 *(-)	0.012 *(-)	0.014 ***(-)	ns

*ns* Not significant, *BL* Buccolingual, *C* Canine, *CEJ* Cementoenamel junction, *CI* Central incisor, *CSR* Canal shape ratio, *LI* Lateral incisor, *LR* Length ratio, *MD* Mesiodistal, *WR* Width ratio

^*^P value < 0.05, ^**^P value < 0.01, ^***^P value < 0.001

CSR demonstrated significant negative correlations with age in the maxillary and mandibular central incisors and mandibular lateral incisors (*p* ≤ 0.05). LR showed limited age association, with significant negative correlations observed only in the maxillary right central incisor and maxillary left lateral incisor (*p* < 0.05).

### Sex and age associated differences (Table 4)

After adjusting for age, according to stepwise linear regression analysis, sex was significantly associated with a limited number of morphometric indices. Females exhibited smaller buccolingual WR at the CEJ in the maxillary right lateral incisor (B = − 0.051, *p* < 0.001), smaller mesiodistal WR at the CEJ in the maxillary left central incisor (B = − 0.037, *p* = 0.033), smaller mesiodistal WR at the mid-root in the mandibular right lateral incisor (B = − 0.032, *p* = 0.028), and smaller CSR at the mid-root in the maxillary left lateral incisor (B = − 0.153, *p* = 0.045). Conversely, females demonstrated larger buccolingual WR (B = 0.019, *p* = 0.029) and CSR values (B = 0.156, *p* = 0.033) at the apical third of the maxillary right central incisor.

**Table 4:** Multiple linear regression models evaluating the associations of sex and age (as independent variables) with morphometric indices, with results presented after adjustment for age. Negative B values indicate lower values in females relative to males

Tooth	Root level	Index	Direction in females	B	SE	β	t	*p*-value
Maxillary right LI	CEJ	WR (BL)	Smaller	−0.051	0.012	−0.365	−4.133	< 0.001
Maxillary left CI	CEJ	WR (MD)	Smaller	−0.037	0.017	−0.214	−2.165	0.033
Mandibular right LI	Mid-root	WR (MD)	Smaller	−0.032	0.014	−0.215	−2.230	0.028
Maxillary left LI	Mid-root	CSR	Smaller	−0.153	0.075	−0.200	−2.035	0.045
Maxillary right CI	Apical third	WR (BL) CSR	Larger Larger	0.019 0.156	0.009 0.072	0.186 0.196	2.216 2.162	0.029 0.033

*BL* Buccolingual, *CEJ* Cementoenamel junction, *CI* Central incisor, *CSR* Canal shape ratio, *LI* Lateral incisor, *MD* Mesiodistal, *WR* Width ratio

Across the stepwise logistic regression models, indices such as mesiodistal WR at the apical third, mesiodistal WR at the CEJ, buccolingual WR at the CEJ, CSR at the CEJ, CSR at the apical third, and mesiodistal WR at the mid‑root were intermittently retained, with corresponding Wald statistics indicating modest explanatory contributions. These findings are presented solely as exploratory indicators of potential sex‑associated morphometric patterns and should not be interpreted as clinically meaningful or as evidence of a validated sex‑prediction model. The full logistic regression coefficients, standard errors, Wald statistics, and p‑values for all retained indices are provided in Supplementary Table S1.

### Age estimation models (Table 5)

**Fig. 3:**
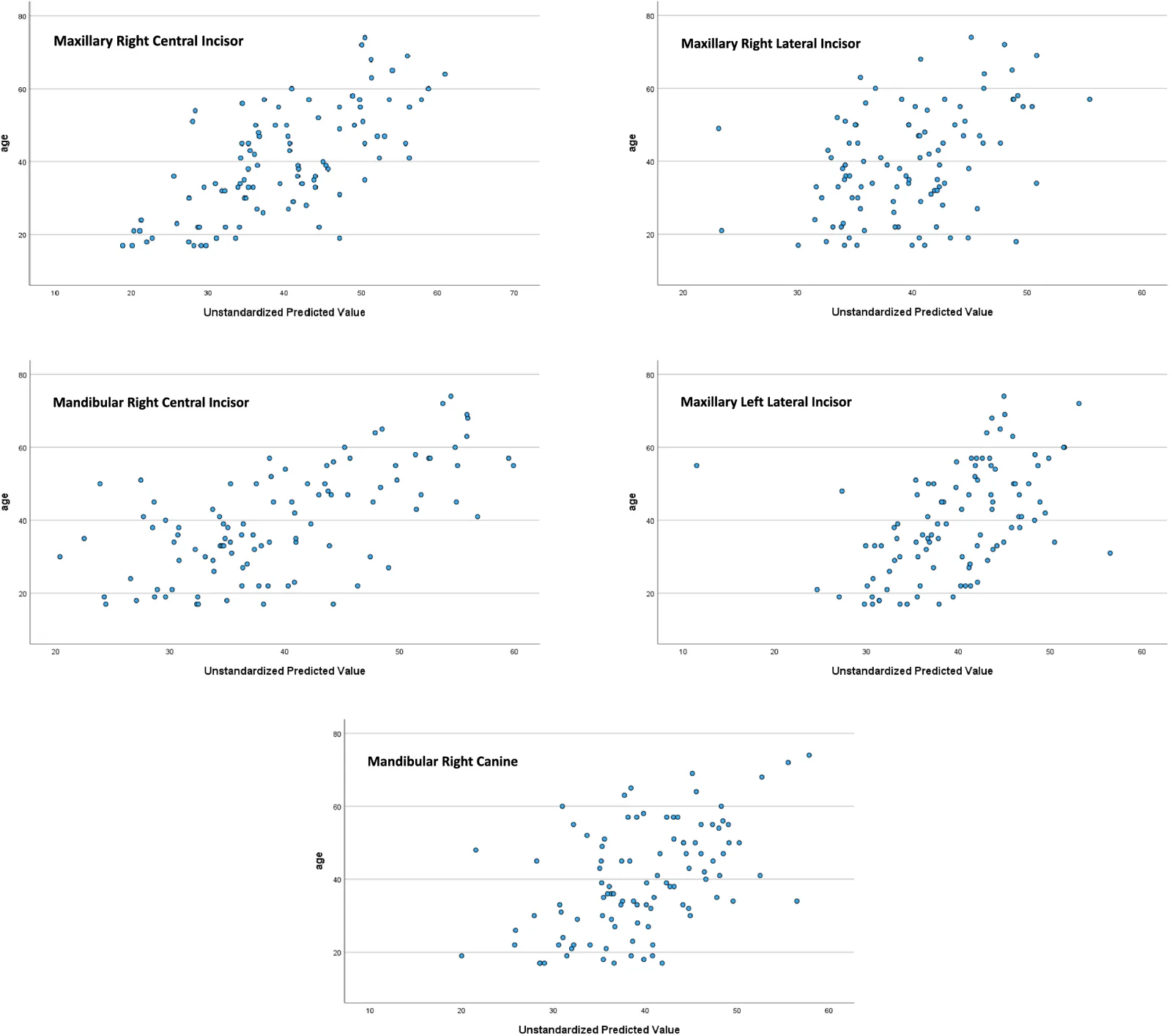
Observed-versus-predicted age scatter plots for selected tooth‑specific multiple linear regression models. Each panel displays the relationship between chronological age (y‑axis) and the unstandardized predicted values generated from the corresponding regression model (x‑axis). The figure includes representative teeth spanning the strongest, moderate, and weakest apparent explanatory performance to illustrate tooth‑specific variability. These plots complement the R², MAE, and RMSE values reported in Table 5 and should be interpreted as exploratory visualizations rather than validated predictive performance

The explanatory performance of morphometric indices for age estimation is summarized in Table 5. No indices were significantly associated with age for the maxillary left canine. The strongest and weakest apparent explanatory performances were observed in the maxillary right central incisor (R² = 0.465, *p* < 0.001) and the mandibular left canine (R² = 0.109, *p* = 0.003), respectively. Overall, buccolingual WR at the CEJ emerged as the most consistently retained and strongest standardized association with age across models (all *p* ≤ 0.05). An exception was observed for the maxillary right canine, which contrasted with the generally weak age‑related associations seen in other canines. This tooth retained multiple indices in the exploratory age‑estimation models, with mesiodistal WR at the apical third demonstrating the largest standardized β (β = 0.567, *p* < 0.001). Clinically meaningful error metrics (mean absolute error and root mean square error) were calculated for each model to complement R² values and provide additional context for apparent model fit. Observed chronological age versus model‑estimated age has been plotted and presented in Fig. 3 for these multiple regressions.

**Table 5:** Multiple linear regression models evaluating exploratory associations between age (dependent variable) and morphometric indices

Tooth	Predictor	B	95% Confidence Interval for B (Lower, Upper)	SE of B	β	*p*-value	*R* ^2^	MAE	RMSE
Maxillary right CI	Constant	72.518	(59.969, 85.068)	6.322	NA	< 0.001	0.465	8.48	10.61
	WR at CEJ (BL)	-105.792	(-148.733, -62.851)	21.633	− 0.421	< 0.001			
	WR at mid-root (BL)	-114.512	(-176.414, -52.610)	31.185	-0.313	< 0.001			
	WR at apical third (MD)	25.217	(0.003, 50.430)	12.702	0.152	0.050			
Maxillary left CI	Constant	75.263	(66.045, 84.480)	4.644	NA	< 0.001	0.407	9.21	11.17
	WR at CEJ (BL)	-106.211	(-153.649, -58.773)	23.901	-0.437	< 0.001			
	WR at apical third (BL)	-88.570	(-152.754, -24.386)	32.339	-0.269	0.007			
Maxillary right LI	Constant	43.351	(29.690, 57.012)	6.883	NA	< 0.001	0.164	10.79	13.27
	WR at CEJ (BL)	-72.991	(-114.784, -31.198)	21.057	-0.322	0.001			
	WR at apical third (MD)	49.709	(9.128, 90.290)	20.447	0.226	0.017			
Maxillary left LI	Constant	61.206	(52.230, 70.182)	4.523	NA	< 0.001	0.231	10.38	12.72
	WR at CEJ (BL)	-147.487	(-203.716, -91.259)	28.331	-0.670	< 0.001			
	CSR at CEJ	11.047	(3.136, 18.958)	3.986	0.357	0.007			
Maxillary right C	Constant	36.943	(18.898, 54.988)	9.090	NA	< 0.001	0.340	9.33	11.78
	WR at mid-root (BL)	-119.074	(-174.372, -63.776)	27.854	-0.449	< 0.001			
	WR at apical third (MD)	114.520	(67.089, 161.951)	23.892	0.567	< 0.001			
	WR at CEJ (MD)	-92.094	(-138.045, -46.142)	23.147	-0.334	< 0.001			
	CSR at apical third	16.741	(5.771, 27.712)	5.526	0.421	0.003			
Mandibular right CI	Constant	68.073	(60.816, 75.331)	3.657	NA	< 0.001	0.405	9.11	11.19
	WR at CEJ (BL)	-135.375	(-168.249, -102.500)	16.566	-0.637	< 0.001			
Mandibular left CI	Constant	56.081	(46.190, 65.973)	4.984	NA	< 0.001	0.452	8.49	10.73
	WR at CEJ (BL)	-114.111	(-144.668, -83.554)	15.396	-0.581	< 0.001			
	WR at mid-root (MD)	40.621	(10.953, 70.289)	14.948	0.213	0.008			
Mandibular right LI	Constant	70.445	(62.226, 78.664)	4.141	NA	< 0.001	0.385	9.32	11.38
	WR at CEJ (BL)	-115.708	(-150.711, -80.705)	17.636	-0.527	< 0.001			
	CSR at apical third	-5.659	(-9.040, -2.277)	1.704	-0.267	0.001			
Mandibular left LI	Constant	75.557	(67.194, 83.919)	4.213	NA	< 0.001	0.446	8.72	10.79
	WR at CEJ (BL)	-130.656	(-165.994, -95.319)	17.805	-0.567	< 0.001			
	CSR at apical third	-6.805	(-10.947, -2.664)	2.087	-0.252	0.002			
Mandibular right C	Constant	66.836	(53.627, 80.046)	6.656	NA	< 0.001	0.258	10.13	12.49
	WR at CEJ (BL)	-161.597	(-219.023, -104.171)	28.934	-0.605	< 0.001			
	CSR at CEJ	8.811	(4.995, 12.627)	1.923	0.496	< 0.001			
Mandibular left C	Constant	69.864	(52.273, 87.456)	8.863	NA	< 0.001	0.109	11.61	13.69
	WR at CEJ (BL)	-73.291	(-120.982, -25.600)	24.029	-0.299	0.003			
	WR at CEJ (MD)	-53.759	(-101.337, -6.180)	23.972	-0.220	0.027			

*B* unstandardized coefficient, *β* standardized coefficient, *p-value* significance of the predictor, *SE of B* standard error of B, *R²* coefficient of determination for the model, - negative correlation with age, + positive correlation with age, *BL* buccolingual, *C* canine, *CEJ* cementoenamel junction, *CI* central incisor, *CSR* canal shape ratio, *LI* lateral incisor, *LR* length ratio, *MAE* mean absolute error, *MD* mesiodistal, *NA* not applicable, *RMSE* root mean square error, *WR* width ratio

## Discussion

The present study investigated the association between root canal morphometric indices of anterior teeth and chronological age and evaluated their explanatory performance for age estimation in an Iranian population using CBCT imaging. A total of 1,200 maxillary and mandibular anterior teeth from 100 individuals were retrospectively analyzed. By applying standardized morphometric ratios rather than absolute measurements, this study aimed to minimize interindividual anatomical variability and enhance the comparability of age-related changes across different teeth and root levels.

The observed inverse associations of canal shape ratio (CSR) and buccolingual width ratios (WRs) with age—particularly in central incisors and mandibular lateral incisors—are consistent with progressive reductions in pulpal dimensions attributed to secondary dentin deposition described in prior volumetric and CBCT-based studies [19–22]. Notably, the relationship between buccolingual WR and age demonstrated level-dependent variability in canines and maxillary lateral incisors, suggesting spatially heterogeneous secondary dentin apposition along the root. Overall, the strongest and most consistent age-related associations were identified in central incisors and mandibular lateral incisors. Among these, the maxillary central incisor exhibited the most robust and consistent relationships across morphometric indices and achieved the highest coefficient of determination (R²) for age estimation. Model complexity varied by tooth, with the number of retained predictors reflecting tooth-specific morphometric relevance rather than an attempt to maximize explained variance. This finding aligns with multiple CBCT-based investigations reporting superior apparent explanatory performance of maxillary central incisors relative to other anterior teeth [21, 23–27]. In particular, Asif et al. demonstrated the strongest correlation coefficients and the lowest mean absolute error values for maxillary central incisors across evaluated tooth types [25].

Among the evaluated morphometric indices, buccolingual width ratio (WR) at the cementoenamel junction (CEJ) exhibited the most robust and consistent associations with age across anterior teeth, with significant correlations identified in 11 of 12 teeth. In incisors, mesiodistal WRs showed positive age associations whenever statistical significance was achieved. When considered together with the consistently negative association between canal shape ratio (CSR) and age, these findings suggest anisotropic secondary dentin deposition, characterized by proportionally greater buccolingual canal constriction relative to mesiodistal dimensions. This pattern indicates that age-related changes in the pulp–dentin complex are not solely reductive but involve direction-specific geometric remodeling. Similar nonuniform patterns of secondary dentin apposition have been reported in prior micro-CT and radiologic studies [28, 29]. Furthermore, buccolingual WR at the CEJ demonstrated the strongest apparent explanatory performance for age estimation across nearly all anterior teeth. This observation is biologically plausible and consistent with the known pattern of radicular cementogenesis. Although cellular cementum apposition continues throughout life, histologic and high-resolution imaging studies indicate that cementum thickening predominantly affects the apical root region, with minimal contribution at cervical and mid-root levels [30, 31]. Consequently, measurements obtained at the CEJ are unlikely to be substantially confounded by cementum deposition and may more accurately reflect age-related regressive changes in canal morphology. Finite element analyses have shown that occlusal loading generates concentrated tensile and compressive stresses in the cervical and sub-cervical regions of teeth, supporting a mechanistic link between functional stress, secondary dentin deposition, and structural remodeling near the CEJ [32–34]. Collectively, these findings suggest that age-related morphometric changes may reflect, at least in part, a biologic adaptation to long-term functional stress distribution along the tooth.

The present study demonstrated marked tooth-specific variability in the strength of inverse associations between age and canal-to-root morphometric ratios. This observation aligns with prior CBCT-based studies of anterior teeth, which consistently report heterogeneous correlation strengths across tooth types despite comparable imaging modalities and analytic approaches [19, 21, 23–25, 35]. Taken together, these convergent findings suggest that inter-tooth differences in age sensitivity primarily reflect biologically driven patterns of secondary dentin deposition rather than methodological variation related to imaging or segmentation.

Although canines have traditionally been favored for dental age estimation because of their larger dimensions, greater pulp volumes, and presumed structural stability [21], the present study demonstrated limited age sensitivity across several canine-related morphometric indices. This finding is consistent with Haghanifar et al., who reported higher apparent model fit for maxillary central incisors (R² = 0.586; standard error of estimate = 7.045) and the lowest performance for maxillary canines (R² = 0.392; standard error of estimate = 8.387) in an Iranian population [27]. Similar limitations in the explanatory power of canine-based models have been documented in volumetric pulp–tooth ratio studies in Turkish and Chinese populations [36, 37]. The observed‑versus‑predicted age plots (Fig. 3) further illustrate these tooth‑specific differences in apparent model fit. Teeth with tighter clustering of points around the regression line demonstrated higher R² values and lower MAE and RMSE, reflecting better explanatory performance. Consistent with this pattern, the maxillary right central incisor showed the strongest apparent fit, whereas the mandibular left canine exhibited the weakest performance. These visual and numerical patterns reinforce that the explanatory strength of the models is driven by tooth-specific morphometric behavior rather than methodological variation. Collectively, these observations suggest that the modest apparent explanatory performance of canines is more likely attributable to biologically driven patterns of secondary dentin deposition rather than methodological factors. In contrast, Dehghani et al. reported reliable age estimation using canine pulp–tooth area ratios in an Iranian cohort [20]. This discrepancy may be explained by fundamental methodological differences, as their analysis relied on two-dimensional panoramic radiography and area-based measurements rather than three-dimensional CBCT-derived morphometric indices.

Maxillary and mandibular anterior teeth were selected for analysis because of their relatively simple root canal anatomy and high retention rates, which enhance measurement reliability [16, 17, 38]. Analyses were restricted to radicular morphometric indices to minimize the confounding effects of externally driven secondary and tertiary dentin deposition and to better isolate age-related changes [39]. Experimental evidence supports this approach: Oi et al. demonstrated that increased masticatory loading is associated with greater secondary dentin deposition beneath functional cusps, disproportionately influencing coronal pulp morphology [28]. In line with this biomechanical rationale, Boedi et al. reported distinct age correlations between coronal and radicular volumetric indices, with radicular measurements exhibiting superior apparent explanatory performance for chronological age estimation [19]. Although voxel size can influence the accuracy of pulp and canal visualization [40, 41], all scans in the present study were obtained using a single CBCT unit with uniform voxel dimensions across participants. Consequently, inter-scan variability due to voxel size was eliminated, and morphometric measurements remained reproducible and reliable. Any minimal influence of voxel size would have been consistent across all teeth and participants and therefore cannot systematically bias the observed age-related associations.

To minimize confounding factors, only individuals with all anterior teeth present were included, enhancing internal validity. However, this restrictive criterion also limited the sample size, highlighting the need for future studies with larger and more diverse cohorts to improve statistical power and generalizability. Although the study was conducted at a dental school in Tehran serving a socioeconomically diverse population, the findings may not fully represent the broader Iranian population and should therefore be interpreted as reflecting this specific regional subpopulation. Since individuals with missing anterior teeth, restorations, attrition, or periodontal changes were excluded, the final sample represents an ideal dentition and may not reflect the broader adult population, particularly older individuals in whom such conditions are common. In addition, the age distribution of the sample was relatively balanced across age groups, with only seven participants older than 60 years and a maximum age of 74 years, indicating that the dataset was not disproportionately weighted toward older individuals. Although the sex distribution was female‑predominant (2:1), sex did not contribute meaningfully to any of the tooth‑specific linear regression models. In each model, sex entered the stepwise procedure but was removed because it did not improve explanatory performance after accounting for morphometric predictors, suggesting that sex did not exert a measurable independent effect on age‑related morphometric variation within this dataset. Overall, these findings should be interpreted within the context of the study population and may not be directly generalizable to populations with different demographic or dental characteristics.

Although each participant contributed measurements from multiple anterior teeth, the analyses were conducted separately for each tooth type, such that each regression model included one observation per participant. Each tooth‑specific regression analysis was therefore based on independent participant‑level observations rather than pooled tooth‑level data. Accordingly, the reported models should be interpreted as exploratory, tooth‑specific association models rather than validated predictive models. Sex was modeled as an independent predictor rather than a dependent variable, and the regression coefficients in Table 4 represent linear associations rather than logistic effects.

Considering the exploratory nature of the tooth‑specific analyses and the large number of morphometric variables evaluated, the reported age associations should be interpreted cautiously, and isolated p‑values should not be considered confirmatory in the absence of formal adjustment for multiple comparisons. In each tooth‑specific model, the statistical significance of independent variables is interpretable only within that model, and cross‑model comparisons of p‑values or effect sizes were not an objective of this study. As a result, no adjustment procedures were applied for comparing effects across different models.

Methodologically, the incorporation of advanced computational approaches, such as artificial intelligence and machine-learning algorithms, may enhance age-estimation performance by capturing complex, nonlinear relationships among morphometric variables. *I*ntegrating morphometric indices with biomechanical modeling and functional loading parameters in future research could further clarify the role of stress-mediated dentinogenesis in dental aging. In addition, larger and more diverse cohorts, external validation strategies, and modern analytical frameworks may facilitate the development of robust age‑estimation models and help elucidate the mechanisms underlying age‑related dentin formation.

## Conclusions

This study found that root canal morphometric indices of anterior teeth exhibit tooth-specific associations with age in an Iranian population. Age-related changes were most pronounced in the incisors, and the buccolingual width ratio at the cementoenamel junction showed the most consistent association with age. Among individual teeth, the maxillary right central incisor demonstrated the strongest apparent model fit. These findings highlight the relevance of level-specific morphometric assessment in CBCT-based evaluations of age-related changes in the dentin–pulp complex.

## Supplementary Information

Supplementary Material 1. Table S1: Multiple logistic regression results for exploratory sex‑associated differences, with sex modeled as the dependent variable.

## Data Availability

All data generated and analyzed in this study are included within the article and its supplementary materials. Additional raw measurement files are available from the corresponding author upon reasonable request.

## Abbreviations

BL: Buccolingual
B: Unstandardized regression coefficient
β: Standardized regression coefficient
CBCT: Cone‑beam computed tomography
CEJ: Cementoenamel junction
CI: Central incisor
CSR: Canal shape ratio
DICOM: Digital Imaging and Communications in Medicine
Df: Degrees of freedom
ICC: Intraclass correlation coefficient
LI: Lateral incisor
LR: Length ratio
MAE: Mean absolute error
MD: Mesiodistal
NA: Not applicable
RMSE: Root mean square error
SD: Standard deviation
SEE: Standard error of the estimate
SPSS: Statistical Package for the Social Sciences
STROBE: Strengthening the Reporting of Observational Studies in Epidemiology
VIF: Variance inflation factor
WR: Width ratio
